# Morphological diversity of sperm: A mini review

**Published:** 2014-04

**Authors:** Seppan Prakash, Elumalai Prithiviraj, Sekar Suresh, Nagella Venkata Lakshmi, Mohanraj Karthik Ganesh, Murugesan Anuradha, Lakshmanan Ganesh, Premavathy Dinesh

**Affiliations:** 1 *Department of Anatomy, Dr. Arcot Lakshmanasamy Mudaliar Postgraduate Institute of Basic Medical Sciences, University of Madras, Taramani Campus, Chennai, India.*

**Keywords:** *Sperm*, *Sperm competition*, *Sperm morphology*, *Evolution*

## Abstract

Sperms are highly specialized cells for delivering DNA from male to the ovum. Incredibly, wide degree of diversity in sperm morphology in their basic structures i.e. head, middle piece and tail is found across species. Differences in terms of overall size of the sperm, shape and number of sperm produced are also incredible. One of the key for this variations or diversity in sperm may be associated with female reproductive tract, sperm competition, testicular size and sperm size and number. Establishing a correlation between sperm morphology and factors influencing them is a phenomenal task. In this mini-review these associations and the anatomical and functional adaptations among different from of sperm cells that have evolved to optimize fertilization success are discussed. Nevertheless, explaining these morphological diversities in sperm cells is a challenging question and it seems that evolutionary biologists have only recently engaged in exploring its links and patterns. From the literatures it seems that there is no causal relationship between sperm size and testicular size, however, the accumulated knowledge do indicates evolution of sperm morphology across species has some associations with female reproductive tract, sperm competition and sperm size and number, however interpreting these results for phylogentic correlations should be approached with caution.

## Introduction

Sperm is a highly specialized cell for delivering DNA from male to the ovum. Sperm cells are produced in testis by a dynamic process known as “spermatogenesis”, this process show great similarity among organisms (1). However, there are extensive variations in sperm morphology across species. Incredibly, wide degree of diversity in sperm morphology in their basic structures i.e. head, middle piece and tail is found across species. Differences in terms of overall size of the sperm, shape and number of sperm produced are also incredible. 

One of the keys to this variations or diversity in sperm may be associated with female reproductive tract, sperm competition, testicular size and sperm size and number. The evolution of sperm in sexual method of reproduction had shown complex developmental processes, leading to extreme species variation in sperm morphology (2-4). Establishing a correlation between sperm morphology and factors influencing them is a phenomenal task. A general adaptive explanation for this diversity is lacking. In this mini-review these associations and the anatomical and functional adaptations among different species that have evolved to optimize fertilization success are discussed.

**Influence of female reproductive tract**

The expectation is justified that sperm morphology should be influenced by female reproductive anatomy and/or the fertilization environment (5, 6). The sperm phenotype comes from enigmatic female choice (7-9). The sperm must operate inside the females' reproductive tract hence; the female reproductive tract can have important effects on sperm performance. The potential importance of female-derived effects in mammals, birds, and insects showing that sperm length is positively associated with the length of the sperm storage organs of females (spermatheca) (3).

Thus, sperm length appears to respond positively or negatively to evolutionary increases in female reproductive tract dimensions (10, 11). Reports also suggest that smaller the sperm more may be the advantage in terms of numbers in competing for fertilization. Across the species the sperm of internally fertilizing species are generally longer than those of externally fertilizing species, where the influence of female reproductive tract is minimal or nil (3, 12).

The variation in survival of sperm in female tract is tremendous across species. Sperm of rat survives for few hours in female tract and in human and monkey sperm survives for few days (13-15). Sperm survival in female reproductive tracts could extent to months in bats or even years in some fishes and reptiles (16-18). This shows the influence of female reproductive tract on sperm survival and their successes; here the role of sperm or middle piece length (mitochondria volume) seems to be negligible. It appears that rationalize understanding of sperm-female co-evolution is challenging.

**Sperm competition**

Sperm competition (sperm from different males compete for fertilization) in polyandry (females mate with more than one male) is recognized as a potent evolutionary force that has led to sexually selected adaptations (9, 19-21). These adaptations either enhance a male’s own sperm competitiveness, or eliminate rival sperm and the whole organism (9). Such adaptations can cause sexually selected sperm with winning architectures over rival sperm (22). Basically, long tail sperm were seen in lower animals than highly evolved species.

There are several theories regarding the evolution of sperm size and sperm competition favoring males with longer sperm, due to their enhanced swimming velocity and therefore competitiveness (23). Studies have reported that sperm cells are on average longer in polyandrous species compared to monandrous species (female mating with single male in a season) (10, 24). The evolutionary explanation for finding adaptive reason that how longer sperm might be more competitive than shorter ones requires inter-disciplinary re-evaluation of accumulated knowledge and input from studies involving fields like physics, biomechanics, hydrodynamics etc. which seems to be mandatory to understand the link between sperm form (length) and function (velocity) and how the selection acts (25). 

**Testicular size**

Literature indicates relationship between sperm competition and the relative size of the testes. The adaptation of sperm competition and the relative size of the testes of an animal were identified across many taxa such as primates, mammals, birds, frogs, bats and fishes (16, 26-31). There is strong evidence that taxa whose mating pattern generates higher levels of sperm competition have evolved relatively larger testes. Thus the risk of sperm competition in which a male is likely to emerge as an adult develop larger testes and produce bigger ejaculates. However, from the literatures it seems that there is no causal relationship between sperm size or length and testicular size (32).

**Sperm size and number**

There is an enormous diversity in shape of the sperm in animal kingdom; such as giant sperm, even longer than the animal itself, as seen in fruit fly (*Drosophila*); where the fly is just half a centimeter long and its sperm length is up to 6centimetres.This indicates that sperm competition and enigmatic female choice lead to decreased sperm number by favoring longer sperm (6). In this case, as the sperm become less abundant, ova become relatively abundant, and competition between males for fertilization success is predicted to weaken, therefore, is expected to be self-limiting (33).

The presence of multi-flagellate sperm and sperm that lack flagella entirely are another form of diversity (34, 35). It appears that potentially a flagellate sperm can be produced in more number and are less costly to produce, both in terms of energy and time when compare to multi-flagellated sperm. Moreover, selection may therefore favour sperm that lost flagellum in monandrous taxa, where a single male available for a group of females, hence, the competition for sperm was absent and where sexual selection is not a factor.

Coming to the mammalian sperm which are characteristically tiny, but vary in length, starting from 28 μm in the porcupine *Hystrixafricaeaustralis* to 349 μm in the honey possum *Tarsipesrostratus *and the average length of sperm in human, monkey and rat are 60 μm, 70 μm and 160 μm, respectively (36). Wide spectrum of variation in size and number do show mild relation to the presence or absence of sexual selection for given species.

## Conclusion

**Concluding remarks**

Nevertheless, explaining these morphological diversities in sperm cells is a challenging question and it seems that evolutionary biologists have only recently engaged in exploring its links and patterns. From the literatures it seems that there is no causal relationship between sperm size and testicular size, however, the accumulated knowledge do indicates evolution of sperm morphology across species has some associations with female reproductive tract, sperm competition and sperm size and number, however interpreting these results for phylogentic correlations should be approached with caution. Further studies with multi-disciplinary approach would through more light on this science. Collective evolutionary adaptability of the male gametes in an organism determines the survival of species and then they go for a fight among themselves for successful coitus.

## References

[1] Bonilla E, Xu EY (2008). Identification and characterization of novel mammalian spermatogenic genes conserved from fly to human. Mol Hum Reprod.

[2] Breed WG (2005). Evolution of the spermatozoon in muroid rodents. J Morphol.

[3] Humphries S, Evans JP, Simmons LW (2008). Sperm competition: linking form to function. BMC Evol Biol.

[4] Werner M, Simmons LW (2008). Insect sperm motility. Biol Rev Camb Philos Soc.

[5] Gomendio M, Roldan ER (1993). Coevolution between male ejaculates and female reproductive biology in eutherian mammals. Proc Biol Sci.

[6] Miller GT, Pitnick S (2002). Sperm-female coevolution in Drosophila. Science.

[7] Keller L, Reeve HK, Why do females mate with multiple males? The sexually selected sperm hypothesis (1995). Adv Stud Behav.

[8] Thornhill R (1983). Cryptic female choice and its implications in the scorpion fly Harpobittacusnigriceps. Am Naturalist.

[9] Birkhead TR, Moller AP (1998). Sperm competition and sexual selection.

[10] Briskie JV, Montgomerie R, Birkhead TR (1997). The evolution of sperm size in birds. Evolution.

[11] Schulte-Hostedde AI, Millar J (2004). Intraspecific variation of testis size and sperm length in the yellow-pine chipmunk (Tamiasamoenus): implications for sperm competition and reproductive success. Behav Eco Soc Biol.

[12] Stockley P, Gage JG, Parker GA, Møller AP (1996). Female reproductive biology and the coevolution of ejaculate characteristics in fish. Biol Sci.

[13] Harper MJK, Nobil E, Neill JD (1994). Gamete and zygote transport. The Physiology of Reproduction.

[14] Ahlgren M (1975). Sperm transport to and survival in the human fallopian tube. Gynecol Invest.

[15] Maria-Elena O, Gloria G, Carmen-Gloria L, Elena H, Enrique V, Horacio B (1995). Croxatto. Sperm Migration through the Female Genital Tract of the New World Monkey Cebus paella. Biol Reprod.

[16] Hosken DJ (1997). Sperm competition in bats. Proc Biol Sci.

[17] Potter H, Kramer CR (2000). Ultrastructural observations on sperm storage in the ovary of the platyfish, Xiphophorus maculatus (Teleostei: poeciliidae): the role of the duct epithelium. J Morphol.

[18] Sever DM, Hamlett WC (2002). Female sperm storage in reptiles. J Exp Zool.

[19] Parker GA (1970). Sperm competition and its evolutionary consequences in the insects. Biol Rev.

[20] Birkhead TR, Møller AP (1992). Sperm competition in birds.

[21] Darwin CR (1871). The descent of man, and selection in relation to sex.

[22] LaMunyon CW, Ward S (1998). Larger sperm outcompete smaller sperm in the nematode Caenorhabditiselegans. Proc Biol Sci.

[23] Briskie JV, Montgomerie R (1992). Sperm size and sperm competition in birds. Proc Biol Sci.

[24] Gage MJG (1994). Associations between body size, mating pattern, testis size and sperm lengths across butterﬂies. Biol Sci.

[25] Fitzpatrick JL, Garcia-Gonzalez F, Evans JP (2010). Linking sperm length and velocity: the importance of intramale variation. Biol Lett.

[26] Short RV (1979). Sex determination and differentiation. Br Med Bull.

[27] Harcourt AH, Harvey PH, Larson SG, Short RV (1981). Testis weight, body weight and breeding system in primates. Nature.

[28] Kenagy GJ, Trombulak SC (1986). Size and function of mammalian testes in relation to body size. J Mammal.

[29] Moller AP, Briskie JV (1995). Extra-pair paternity, sperm competition and the evolution of testis size in birds. Behavio Ecol Socio Biol.

[30] Jennions MD, Passmore NI (1993). Sperm competition in frogs: testis size and a sterile male experiment on Chiromantis xerampelina (Rhacophoridae). Biol J Linnean Soc.

[31] Stockley P, Gage JG, Parker GA, Møller AP (1997). Sperm competition in fishes: the evolution of testis size and ejaculate characteristics. Am Naturalist.

[32] Gage MJ, Freckleton RP (2003). Relative testis size and sperm morphometry across mammals: no evidence for an association between sperm competition and sperm length. Proc Biol Sci.

[33] Bjork A, Pitnick S (2006). Intensity of sexual selection along the anisogamy-isogamy continuum. Nature.

[34] Bacetti B, Dallai R (1977). The first multi-flagellate spermatozoa in the animal kingdom discovered in Mastotermes darwiniensis. CR Acad Sci Hebd Seances Acad Sci D.

[35] Morrow EH (2004). How the sperm lost its tail: the evolution of aflagellate sperm. Biol Rev Camb Philos Soc.

[36] Gage MJ (1998). Mammalian sperm morphometry. Proc Biol Sci.

